# Experiences, awareness, perceptions and attitudes of women and girls towards menstrual hygiene management and safe menstrual products in Pakistan

**DOI:** 10.3389/fpubh.2023.1242169

**Published:** 2023-09-07

**Authors:** Madeeha Malik, Ayisha Hashmi, Azhar Hussain, Waleed Khan, Nabia Jahangir, Anam Malik, Naima Ansari

**Affiliations:** ^1^Cyntax Health Projects, Contract Research Organization & Corporate Firm, Islamabad, Pakistan; ^2^Pak-Austria Fachhochschule: Institute of Applied Sciences and Technology, Haripur, Pakistan; ^3^Islamabad Women Chamber of Commerce & Industry, Islamabad, Pakistan

**Keywords:** menstrual health, menstrual hygiene management, organic menstrual products, sustainable development goals, Islamabad, Rawalpindi Pakistan

## Abstract

**Background:**

The taboo of menstruation, lack of discussion on puberty, misinformation and poor awareness on menstrual hygiene management and limited access to safe menstrual products can negatively impact the physical and mental health of women and girls residing in low middle income countries.

**Aim:**

The aim of the study was to explore the experiences, awareness, perceptions and attitudes of women and girls towards menstrual hygiene management in Pakistan. Moreover, the study also assessed consumer satisfaction towards locally manufactured organic menstrual products.

**Methods:**

A descriptive cross-sectional study design was used with a sample of 400 women and girls selected through convenience sampling from high schools, universities, outpatient department and gynaecological clinics located in 2 cities, i.e., Islamabad and Rawalpindi, Pakistan. A pre-structured questionnaire was used to explore experiences, awareness, perceptions and attitudes of women and girls towards menstrual hygiene management. Moreover, each respondent was provided with sample of locally designed and manufactured organic menstrual hygiene & wellness kit by a group of women researchers named “FemPure” including organic sanitary pads, feminine wellness mist and feminine wellness wash. The respondents were asked to use the products and a telephonic follow-up was conducted to assess consumer satisfaction for the products after a period of 1 month. Data was analyzed statistically using SPSS 21.

**Results:**

The results of the study reported that 86.2% (*n* = 345) of the respondents had normal periods. Out of 400 respondents, 58.5% (*n* = 234) knew about any health conditions related to abnormal menstrual cycle while 88.3% (*n* = 353) were aware of female menstrual hygiene. Majority of the respondents 78.7% (*n* = 315) felt ashamed while buying sanitary pads. Out of 400 respondents, 5.4% (*n* = 22) were interested in getting awareness regarding menstrual hygiene. The results of the study showed that all the respondents (100%, *n* = 400) were satisfied with FemPure organic menstrual products.

**Conclusion:**

The study concluded that majority of women and girls faced menstrual hygiene issues during and after the cycle and were eager to receive information on MHM and use organic rash free menstrual wellness products which could be ordered via mobile app. The respondents were satisfied after the use of FemPure organic menstrual products.

## Introduction

Menstruation is a biological process that occurs in females during their reproductive years as a result of hormonal fluctuations in the body. However, despite its regular occurrence, menstruation is often considered as a taboo in many cultures and societies (1). This taboo nature of menstruation, lack of discussion on puberty and misinformation from peers leads to inadequate knowledge and poor awareness on menstrual hygiene management among young girls, which can negatively impact the physical and mental health of women and girls. The normal menstruation cycle becomes a subject of shame and embarrassment for women and girls in such societies (2). This further increases the chances of unhygienic practices leading to severe reproductive and urinary tract infections (3). Menstrual hygiene management (MHM) refers to the practices and products used by women and girls to manage their menstrual hygiene during their menstruation period. It includes the use of menstrual products, personal hygiene practices, and waste management. However, access to proper MHM is often limited due to various factors, such as lack of access to affordable menstrual products, inadequate sanitation facilities, and social stigma surrounding menstruation (4). A systematic review reported that girls in majority of the developing countries have gaps in knowledge regarding menstrual hygiene and fallacious beliefs about menstruation (5). In addition to poor knowledge towards menstruation, women and girls in low- and middle income countries are facing period poverty and deprived of available resources related to menstrual hygiene. There is a lack of structured education in schools, poor cultural acceptance of menstrual hygiene products, low economic resources, inadequate water, sanitation and hygiene facilities and poor access to safe menstrual products leading to period poverty. Inability to manage menstrual hygiene adequately results in absenteeism, anxiety and reduced performance of women and girls (6).

Access to safe, affordable menstrual products is one of the primary challenges towards proper menstrual hygiene management. In many low-income countries, safe menstrual products are either unaffordable or unavailable. It has been estimated that 12.3 to 75% of girls residing in developing countries cannot access or afford clean sanitary materials, and they use low-quality products such as new or old clothes, cotton wool, toilet paper, underwear alone, and sponges (7). There are a few options available to manage menstruation in developing countries with single use sanitary pads commonly being used. These single use pads release volatile organic compounds (VOCs) and endocrine-disrupting chemicals leading to health hazards (8). Around 150,000 tons of sanitary napkin waste is generated every year and these single-use sanitary napkins are composed of more than 90% plastic which do not undergo biodegradation, exhausting landfill sites, for another 700–800 years (9). The increased access to suitable products during menstruation allow women and girls to continue their daily activities without fear of leakage. The major consideration for menstrual products include comfortability, absorbing capacity and no skin irritation and allergies from product use. Other considerations for choice of product include cost, access and easy disposal (5).

In Pakistan, menstrual hygiene management is a significant problem, particularly in low-income communities, where women and girls often lack access to basic menstrual hygiene products. According to UNICEF, 44% of the girls do not have access to basic menstrual hygiene facilities at home, their workplace or school. Approximately, 49% of the women and girls had no knowledge of menstruation prior to their first period. The major source of information are mothers followed by teachers for few of the girls. More than two-thirds of Pakistani women and girls cannot afford sanitary pads whereas the remaining one third use sanitary pads of varying quality (10). The issue of period poverty in Pakistan is further increased by cultural taboos surrounding menstruation, which leads to lack of education and awareness about menstrual hygiene. Girls are often not taught about menstruation in school, and many families consider it as a taboo in the country. The lack of access to safe sanitary pads can have significant negative impacts on women and girls’ health, education, and overall well-being (11). Access to safe menstrual hygiene products along with education is one of the important targets for sustainable development goal 3 health and well-being and 4 quality education as well as production of safe sanitary biodegradable products is an important target for SDG 12 responsible consumption and production and SDG 13 climate action (12). The literature shows that majority of the women products are being designed and manufactured by men around the globe. However, now-a-days many women from different countries are getting involved in this as well, e.g., Eco Femme is a women-led social enterprise founded based in Tamil Nadu, India, which produce and sell washable cloth pads, provide menstrual health education and open dialogs on menstruation all along the way. Similarly, a group of women healthcare researchers and entrepreneurs from Pakistan designed and manufactured organic menstrual hygiene & wellness kit named “FemPure” including organic sanitary pads, feminine wellness mist and feminine wellness wash for women with aim to increase access to safe and cost effective menstrual products which could be ordered using a mobile application along with awareness to break the taboo and shame attached to menstruation in developing countries including Pakistan. Assessing the experiences, awareness, behaviors, perceptions and attitudes towards menstrual hygiene management and safe menstrual products of women and girls is crucial to understand the gaps to improve hygienic practices and reduce period poverty as well as for providing alternate solutions is important. Therefore, this study was conducted to assess the experiences, behaviors, awareness, perceptions and attitudes of women and girls towards menstrual hygiene management using safe menstrual products and satisfaction after using FemPure Organic Menstrual Products in Pakistan.

## Methodology

### Study design and settings

A descriptive cross-sectional study design was used. Data was collected from January to March 2023 from women attending public and private outpatient departments, gynaecological clinics, high schools and universities located in urban areas of the two cities, i.e., Islamabad and Rawalpindi, Pakistan.

### Study population and sampling

Study population included girls and women in the reproductive age experiencing menstruation, i.e., 13–50 years. The sample size was calculated using Rao soft at 95% confidence interval and 5% margin of error which came out to be 400. The respondents were selected through convenience sampling.

### Ethical approval

Study approval was obtained from the Ethical Research Committee of Pak-Austria Fachhochschule University, Haripur Pakistan (Ref. No. /ERC/2023/120). Written informed consent was also taken from respondents before collecting data and providing samples of FemPure Organic Menstrual products.

### Data collection tool

Two structured questionnaires were developed after extensive literature review. The first questionnaire included a section on demographic and contact information followed by 12 questions on experiences regarding menstruation, two questions on awareness of menstrual health and hygiene, two questions regarding perceptions on MHM and three questions on attitudes towards use of menstrual products. The second questionnaire was a consumer survey on satisfaction with FemPure organic menstrual products which included four questions on satisfaction of women and girls towards FemPure Organic Menstrual Products. Pilot testing was conducted on 10% of sample size. The Cronbach alpha value for the first questionnaire was found to be 0.8 and 0.79 for the second questionnaire which indicated the reliability of questionnaire.

## Data collection procedure and data analysis

The questionnaire on experiences, awareness, perceptions and attitudes regarding menstrual health and hygiene management were self-administered to respondents by 6 female data collectors. Each data collector distributed samples of FemPure Organic Menstrual products including a pack of 8 sanitary pads, one 50 mL organic feminine wellness mist and one 15 mL organic feminine wellness mist to each respondent after data collection. The respondents were also provided with information leaflets on directions to use the product. Respondents were instructed to use the products during their menstrual cycle as well as after the menstrual cycle. The data on consumer satisfaction questionnaire was collected after a time period of 1 month from the first data collection through a telephonic follow-up. Data was cleaned, coded and statistically analyzed in SPSS 21. Descriptive statistics comprising of frequencies and percentages were determined. Chi square test (*p* ≤ 0.05) was applied to determine the association between demographic variables and experiences, awareness, perceptions and attitudes of women and girls regarding menstrual health and hygiene management.

## Results

### Demographic characteristics

Out of 400 females, 61.7% (*n* = 247) were married and 38.3% (*n* = 153) were unmarried. Respondents were in the age groups were 13 to 18 years (95%, n = 23.7), 18 to 29 years (35.5%, *n* = 142), 30 to 39 years (31.2%, *n* = 125), 40 to 49 years (6.4%, *n* = 26) and 50+ years (3.2%, *n* = 12). Regarding qualification, 9.7% (*n* = 39) had matric degree, 17.2% (*n* = 69) had intermediate degree, 36.6% (*n* = 146) had bachelor’s degree, 34.4% (*n* = 137) had master’s degree while 2.2% were PhD’s. Out of 400 respondents, 47.8% (*n* = 191) were students, 14.1% (*n* = 56) were housewives and 38.1% (*n* = 153) were working women. A detailed description is given in Table 1.

**Table 1:** Demographic characteristics.

Demographics		*n* (%)
Marital status	Married	247 (61.7)
	Unmarried	153 (38.3)
Age groups	13–18	95 (23.7)
	18–29	142 (35.5)
	30–39	125 (31.2)
	40–49	26 (6.4)
	50 and above	12 (3.2)
Education	Matric	39 (9.7)
	Intermediate	69 (17.2)
	Bachelors	146 (36.6)
	Masters	137 (34.4)
	PhD	9 (2.2)
Occupation	Student	191 (47.8)
	Housewife	56 (14.1)
	Working women	153 (38.1)

### Experiences regarding menstruation

Out of 400 respondents, 86.2% (*n* = 345) had normal periods whereas majority of the respondents cycle lasted 3–8 days (92.6%, *n* = 370), followed by 2 days (4%, *n* = 16) and 1 day (3.4%, *n* = 14). Respondents during periods faced different problems such as: Cramps (16%, *n* = 64), body pain (9.6%, *n* = 38), weakness (5.3%, *n* = 21), dizziness (5.3%, *n* = 22) and majority had all of the above mentioned problems (59.6%, *n* = 238). Most of the respondents suffered from leukorrhea (51.6%, *n* = 206), Pre-Menstrual Syndrome (55.3%, *n* = 221) and vaginal irritation during periods (54.3%, *n* = 217). Almost half of them, 51.1% (*n* = 204) felt bad vaginal odor during periods and 25.5% (*n* = 102) felt bad vaginal odor without periods (25.5%, *n* = 102). A detailed description is given in Table 2.

**Table 2:** Experiences regarding menstruation.

Questions		*n* (%)
Are your periods normal?	Yes	345 (86.2)
	No	55 (13.8)
How long your period cycles last generally?	1 day	14 (3.4)
	2 days	16 (4)
	3–8 days	370 (92.6)
	2 weeks	0
	More than 2 weeks	0
If periods are delayed, how many days?	Late for 15 days	90 (22.6)
	Late for 1 month	15 (3.7)
	Late for 3 months	13 (3.3)
	Late for more than 3 months	11 (2.7)
	Periods are not delayed	271 (67.7)
Do you feel the following during periods?	Cramps	64 (16)
	Body pain	38 (9.6)
	Weakness	21 (5.3)
	Dizziness	22 (5.3)
	All of the above	238 (59.6)
	Do not know	17 (4.3)
Behavioral changes observed during the menstrual cycle?	Irritated	47 (11.8)
	Craving for different foods	17 (4.3)
	Mood swings	103 (25.8)
	All of the above	224 (55.9)
	Do not know	9 (2.2)
Do you face problems of Leukorrhea?	Yes	206 (51.6)
	No	133 (33.3)
	Do not know	61 (15.1)
Do you experience Pre-Menstrual Syndrome?	Yes	221 (55.3)
	No	94 (23.4)
	Maybe	85 (21.3)
Do you take any pain killers during periods?	Yes	149 (37.2)
	No	213 (53.2)
	Maybe	38 (9.6)
Do you feel vaginal irritation during periods?	Yes	217 (54.3)
	No	183 (45.7)
Do you feel vaginal irritation generally without periods?	Yes	102 (25.5)
	No	298 (74.5)
Do you feel bad vaginal odor during periods?	Yes	204 (51.1)
	No	196 (48.9)
Do you feel bad vaginal odor without periods?	Yes	102 (25.5)
	No	298 (74.5)

### Awareness regarding menstrual health

Out of 400 respondents, 58.5% (*n* = 234) knew about any health conditions related to abnormal menstrual cycle while 88.3% (*n* = 353) were aware of female menstrual hygiene (Table 3).

**Table 3:** Awareness regarding menstruation.

Questions		*n* (%)
Do you know any health conditions related to abnormal menstrual cycle?	Yes	234 (58.5)
	No	166 (41.5)
Are you aware of female menstrual hygiene?	Yes	353 (88.3)
	No	47 (11.7)

### Perceptions regarding menstrual hygiene management

The results of the current study showed that majority of the respondents (98.9%, *n* = 396) were of the view that there was a need of availability of proper menstrual health in our society without any shame. Majority of the respondents 78.7% (*n* = 315) felt ashamed while buying sanitary pads (Table 4).

**Table 4:** Perceptions regarding menstrual hygiene management.

Questions		*n* (%)
Do you think we need proper menstrual health availability in our society without any shame?	Yes	396 (98.9)
	No	4 (1.1)
Do you feel ashamed buying sanitary pads?	Yes	85 (21.3)
	No	315 (78.7)

### Attitudes towards using menstrual products

The results of the present study highlighted that reasons for using a particular brand of sanitary brands by women included: low cost (3.2%, *n* = 13), extra-long (19.1%, *n* = 76), more absorbent (23.4%, *n* = 94), no rash (19.1%, *n* = 76), while majority (35.1%, *n* = 141) were interested in all mentioned properties of a sanitary pad. Out of 400 respondents, 5.4% (*n* = 22) were interested in getting awareness regarding menstrual hygiene, 7.6% (*n* = 30) in buying organic feminine wash, 17.4% (*n* = 70) in buying rash less sanitary pads, 1.2% (*n* = 5) in liberty to order menstrual products, 4.3% (*n* = 17) placing orders through app for home delivery while majority 64.1% (*n* = 256) were in interested in all of the above mentioned features. A detailed description is given in Table 5.

**Table 5:** Attitudes towards using menstrual products.

Questions		*n* (%)
Why are you using a particular brand of sanitary pad?	Low cost	13 (3.2)
	Extra long	76 (19.1)
	More absorbance	94 (23.4)
	No rash	76 (19.1)
	All of the above	141 (35.1)
If you are offered the following product & serums for safe menstruation, how much you would be interested?	Awareness regarding menstrual hygiene	22 (5.4)
	Organic feminine wash	30 (7.6)
	Rash less sanitary pads	70 (17.4)
	Liberty to order menstrual products	5 (1.2)
	Through app for home delivery	17 (4.3)
	All of them	256 (64.1)
If you are provided with safe organic menstrual hygiene kit including (pads, wash, mist) in PKR 1500 with free home delivery. Would you be interested to purchase?	Yes	265 (66.3)
	No	135 (33.7)

### Satisfaction towards FemPure organic menstrual products

The results of the study showed that all the respondents (100%, *n* = 400) were satisfied with FemPure organic menstrual products. Out of 400 respondents, the most attractive feature of the products found were: quality (7.5%, *n* = 30), price (2.5%, *n* = 10), packaging (3.75%, *n* = 15), information leaflet (2.5%, *n* = 10), availability of range of MHM products (3.75%, *n* = 15) and all of the above features (73.7%, *n* = 295). All of the respondents (100%, *n* = 400) were of the view that they would recommend these products to other women and girls. A detailed description is given in Table 6.

**Table 6:** Satisfaction towards FemPure organic menstrual products.

Questions		*n* (%)
Are you satisfied with the FemPure products provided?	Yes	400 (100)
	No	0
What was the most attractive feature of the products?	Quality	30 (7.5)
	Effectiveness	25 (6.25)
	Price	10 (2.5)
	Packaging	15 (3.75)
	Menstrual hygiene information leaflet	10 (2.5)
	Availability of range of organic MHM products	15 (3.75)
	Placing order through app	
	All of the above	295 (73.7)
Would you recommend these products to other women and girls?	Yes	400 (100)
	No	0

Chi-square test (*p* ≥ 0.05) was used to find association among different variables. Significant association (*p* ≥ 0.05) was found in terms of experience and awareness linked to marital status, age group, occupation and education level. Married women, those in age group of 40 and above and working women relatively had regular periods and face less problems during their cycle. Moreover, women with better education had more awareness regarding menstrual health & hygiene (Table 7).

**Table 7:** Comparison of experience, awareness, perceptions and attitude towards menstrual hygiene and products in terms of demographic characteristics.

No	Indicators	Experience		Awareness		Perceptions		Attitude	
		*p* value*	*p* value**	*p* value*	*p* value**	*p* value*	*p* value**	*p* value*	*p* value**
1	Marital status	0.001*	0.001**	0.092	0.194	0.213	0.394	0.087	0.522
2	Age group	0.021*	0.001**	0.923	0.207	0.065	0.845	0.092	0.869
3	Education level	0.082	0.290	0.003*	0.001**	0.712	0.921	0.423	0.291
4	Occupation	0.042*	0.001**	0.090	0.396	0.621	0.238	0.641	0.461

Chi-square test; *significant (*p* ≥ 0.05); ** significant (*p* ≥ 0.1).

## Discussion

Throughout the world, millions of women and girls lack access to safe and appropriate menstrual products, as well as education and information about menstrual health management practices (13). This leads to a high risk of developing infections and other health issues as well as increased stigma and discrimination during their menstruation period (14). The results of the present study showed that majority of the respondents had normal menstrual cycles. However, majority of the respondents suffered from menstrual symptoms in moderate to severe intensity including cramps, body pain, weakness, and dizziness. Similar results were observed in a study conducted in Australia where menstrual symptoms were regularly experienced by women (15). The study showed that more than half of the respondents suffered from leukorrhea, pre-menstrual syndrome and vaginal irritation during their menstrual cycle. Almost half of the respondents suffered from issues such as bad vaginal odor during menstruation and a quarter suffered from bad vaginal odor even afterwards. The findings were inline with a study conducted in India where rural married women suffered from similar symptoms (16).

Lack of knowledge about menstrual health and poor awareness of effective hygiene management practices can lead to increased gynaecological morbidity among women and girls as well as poor health related quality of life (17). The results of the current study showed only half of the respondents knew about any health conditions related to abnormal menstrual cycle while majority were aware of female menstrual hygiene. Similar results were observed in a study conducted in India where women were aware of menstrual hygiene practices (18). The results of the current study showed that majority of the respondents were of the view that there was a need proper menstrual health services in our society without any shame as majority of the respondents felt ashamed while buying sanitary pads. The findings are in line with a study conducted in Indonesia where young girls felt embarrassed while buying menstrual products (19).

Personal preference, cultural norms, access to resources, and individual experiences of women and girls are few of the factors behind selection of menstrual products (20). The results of the present study highlighted that most of the respondents preferred sanitary pads that were of low cost, extra-long, more absorbent, no rash while majority were interested in all mentioned properties of a sanitary pad. A study conducted in Nepal reported that women consumers preferred those brands of sanitary pads which provided extra-long, high absorbance and soft cotton pads (21). Majority of the women were of the view that they were interested in receiving awareness regarding menstrual hygiene, in buying organic feminine wash, in buying rash less sanitary pads, liberty to order menstrual products and placing orders through app for home delivery. The results are in line with a study conducted in India where women were of the view that they prefer product brands which provide information on menstrual hygiene and are easy to use (22).

Providing access to safe and high-quality menstrual health products and information on menstrual health can lead to reduction in side effects due to use of traditional products as well as improved utilization of menstrual hygiene products. The results of the study showed that all the respondents were satisfied with FemPure organic menstrual products. Majority of the respondents were of the view that the most attractive feature of the products was quality, price, packaging, information leaflet and availability of range of MHM products. All the respondents were of the view that they would recommend these products to other women and girls. Similar results were observed in a study conducted in America where women preferred organic and green menstrual products which were biodegradable and safe (23).

The results of the study showed that significant associations were found in terms of experience and awareness linked to marital status, age group, occupation and education level. Married women, those in age group of 40 and above and working women relatively had regular periods and faced less problems during their cycle. Moreover, women with better education had more awareness regarding menstrual health & hygiene. The results are in line with studies conducted in India where educated women had better knowledge regarding menstrual health and hygiene as compared to other women (24, 25).

## Study limitation

The results of the study may not be generalized to whole country as data has been collected from two cities only. Moreover, the respondents were residing in the urban areas and the situation in rural areas might be different. Furthermore, menstruation is still considered as a taboo to be discussed even in the urban areas owed to cultural issues due to which few respondents were reluctant to talk about few aspects openly and their opinions might not reflect their true feelings.

## Conclusion

The study concluded that majority of women and girls faced menstrual hygiene issues during and after the cycle and were eager to receive information on MHM and use products which are organic, rash free and could be ordered via mobile app. Majority of the respondents were of the view that the most attractive feature of the products was quality, price, packaging, information leaflet and availability of range of MHM products. There is a need to improve access to safe and hygienic menstrual products for women and girls along with provision of accurate information on menstrual health. The use of technology in form of mobile apps for women and girls for ordering menstrual products can serve as a means of improving access to products and reducing the stigma in buying the products. UNICEF and WaterAid has extensively worked on training and advocacy of MHM in Pakistan with a plan to introduce women-friendly sanitation facilities in every public building in the next 5 years. However, more National level awareness programs on menstrual health and hygiene for women and girls in educational institutions should be designed to reduce the stigma in discussing the health issues related to menstrual and reproductive health.

## Data availability statement

The original contributions presented in the study are included in the article/supplementary material, further inquiries can be directed to the corresponding author.

## Ethics statement

The studies involving human participants were reviewed and approved by the Ethical Research Committee of PakAustria Fachhochschule University, Haripur Pakistan (Ref. No. /ERC/2023/120). Written informed consent was also taken from respondents before collecting data and providing samples of FemPure Organic Menstrual products.

## Author contributions

MM and AyH conceived designed the study, conducted research, provided research materials, provided logistic support, and analyzed and interpreted data. WK, NJ, and AM collected data. NA and AzH wrote initial draft of the article and analyzed and interpreted data. All authors contributed to the article and approved the submitted version.

## Conflict of interest

The authors declare that the research was conducted in the absence of any commercial or financial relationships that could be construed as a potential conflict of interest.

## Publisher’s note

All claims expressed in this article are solely those of the authors and do not necessarily represent those of their affiliated organizations, or those of the publisher, the editors and the reviewers. Any product that may be evaluated in this article, or claim that may be made by its manufacturer, is not guaranteed or endorsed by the publisher.
